# Controlling cyanobacterial harmful blooms in freshwater ecosystems

**DOI:** 10.1111/1751-7915.12725

**Published:** 2017-06-21

**Authors:** Hans W. Paerl

**Affiliations:** ^1^ Institute of Marine Sciences University of North Carolina at Chapel Hill Morehead City NC 28557 USA

## Abstract

Cyanobacteria's long evolutionary history has enabled them to adapt to geochemical and climatic changes, and more recent human and climatic modifications of aquatic ecosystems, including nutrient over‐enrichment, hydrologic modifications, and global warming. Harmful (toxic, hypoxia‐generating, food web altering) cyanobacterial bloom (CyanoHAB) genera are controlled by the synergistic effects of nutrient (nitrogen and phosphorus) supplies, light, temperature, water residence/flushing times, and biotic interactions. Accordingly, mitigation strategies are focused on manipulating these dynamic factors. Strategies based on physical, chemical (algaecide) and biological manipulations can be effective in reducing CyanoHABs. However, these strategies should invariably be accompanied by nutrient (both nitrogen and phosphorus in most cases) input reductions to ensure long‐term success and sustainability. While the applicability and feasibility of various controls and management approaches is focused on freshwater ecosystems, they will also be applicable to estuarine and coastal ecosystems. In order to ensure long‐term control of CyanoHABs, these strategies should be adaptive to climatic variability and change, because nutrient‐CyanoHAB thresholds will likely be altered in a climatically more‐extreme world.

## Introduction

Cyanobacteria are among the most ancient phototrophs on Earth, having appeared over 2 billion years ago. They ‘invented’ oxygenic photosynthesis, a quantum step in biogeochemical evolution that has shaped the Earth's modern‐day oxic biosphere (Schopf, 2000). Their long evolutionary history has served them well as they have experienced and adapted to myriad climatic and geochemical extremes. They possess heat and desiccation‐tolerant resting cells, sheaths and capsules, photoprotective cellular pigments, and can adjust their buoyancy to optimize growth and their position in the water column in response to irradiance and nutrient gradients (Potts and Whitton, 2000; Reynolds, 2006). They have also developed a wide array of physiological adaptations to periodic nutrient deplete conditions, including the ability to convert or ‘fix’ atmospheric nitrogen (N_2_) into biologically available ammonia, sequester (by chelation) iron, store phosphorus, nitrogen and other essential nutrients (Reynolds, 2006), and produce metabolites that enhance their ability to counter potentially adverse environmental conditions, including photo‐oxidation, and provide protective and adaptive functions (Huisman *et al*., 2005; Paerl and Otten, 2013). Lastly, cyanobacteria have mutualistic and symbiotic associations with other microbes, plants and animals that ensure their survival (Paerl and Millie, 1996) in environments too hostile for individual members to cope with (Paerl, 1988).

**Figure 1:**
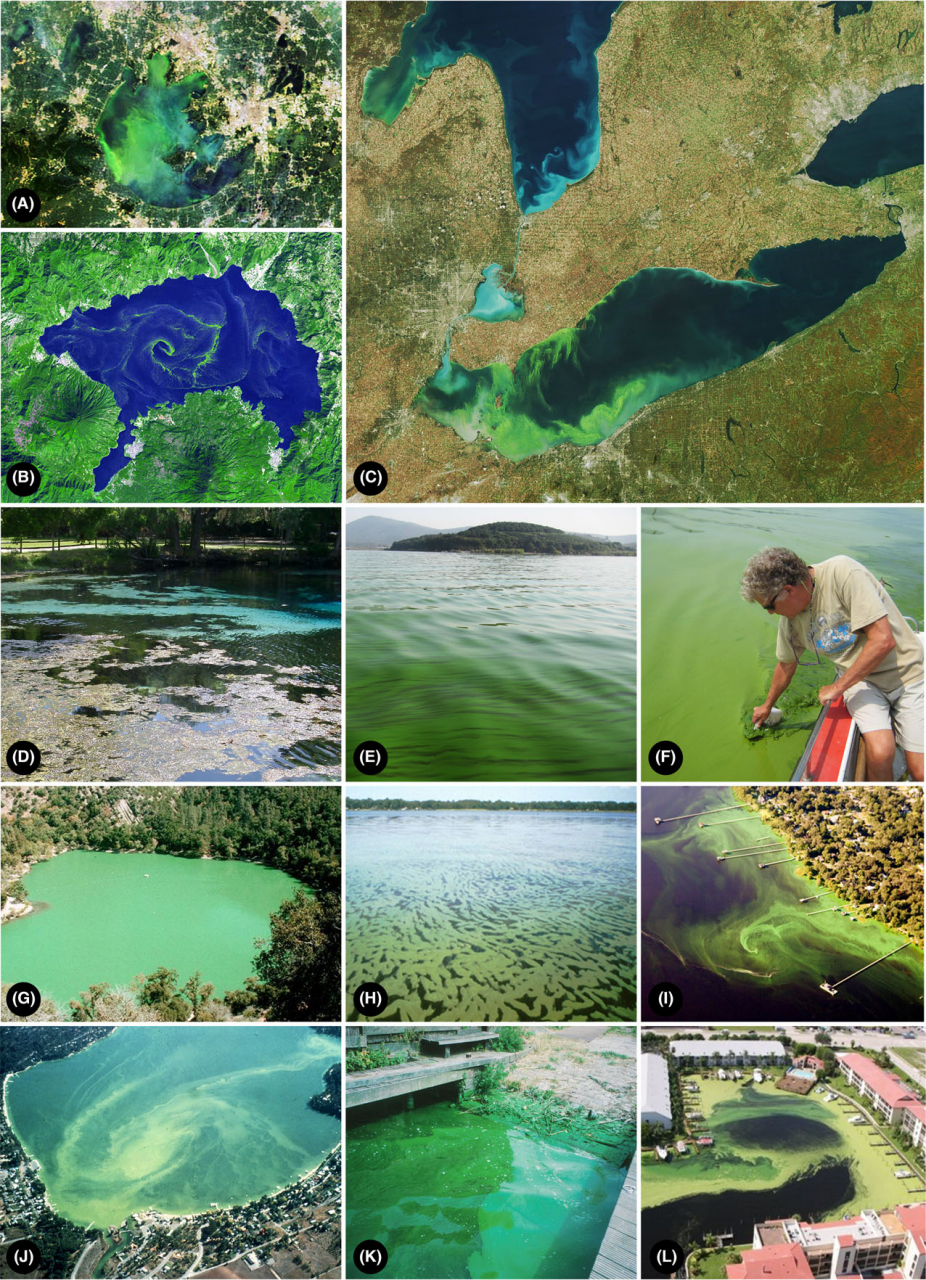
Examples of globally distributed harmful cyanobacterial blooms (CyanoHABs). A. Lake Taihu (NASA MODIS), China. B. Lake Atitlan, Guatamala (NASA Earth Observatory). C. Lake Erie, USA–Canada (NASA MODIS). D. Ichetucknee Springs, Florida, USA (H. Paerl). E and F. Lake Taihu, China (H. Paerl). G. Zaca Lake, California, USA (A. Wilson). H. and I. St. Johns River, Florida (J. Burns). J. Liberty Lake, Washington, USA (Liberty Lake Sewer and Water District). K. Canal near Haarlem, the Netherlands (H. Paerl), L. Lagoon near St. Lucie River, Florida (Ft. Pierce News Tribune).

**Figure 2:**
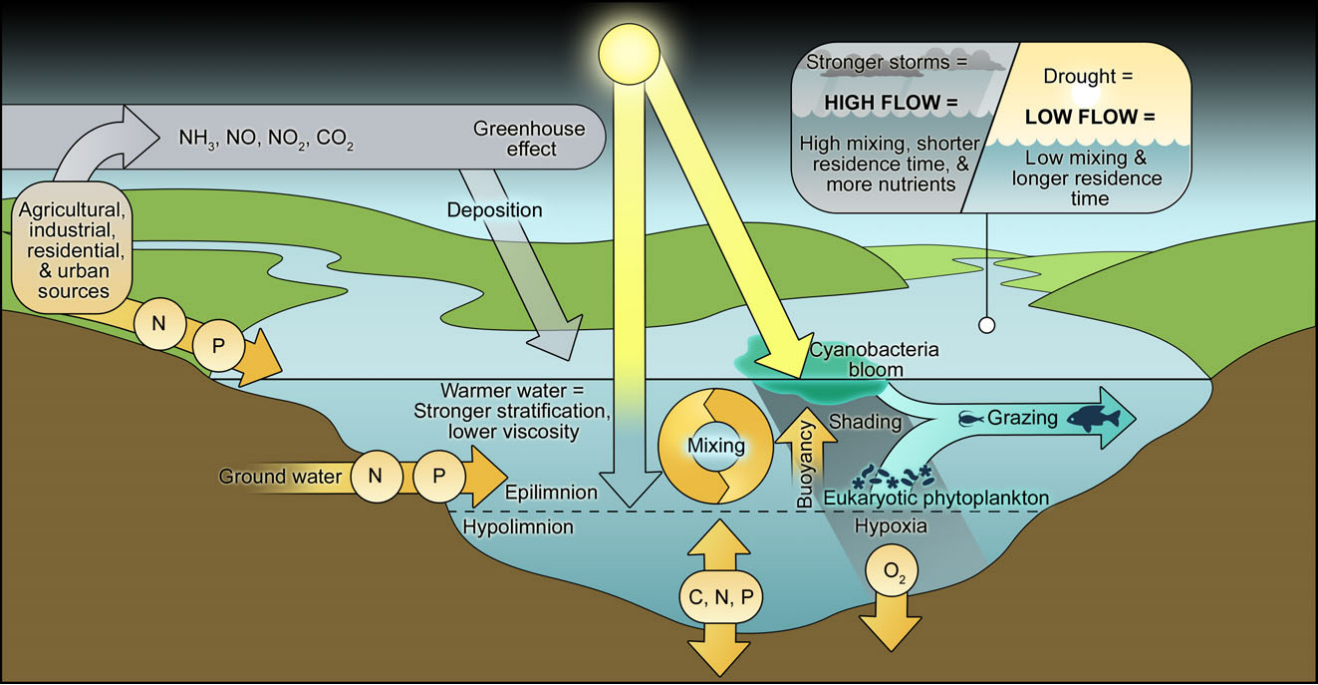
Conceptual diagram, illustrating multiple interacting environmental factors controlling harmful cyanobacterial blooms. Figure adapted from Paerl *et al*. (2011).

These ecophysiological capabilities also enable cyanobacteria to exploit human and climatically driven environmental changes as harmful (toxic, hypoxia‐generating, food web altering and aesthetically undesirable) water blooms or CyanoHABs, a major threat to the use, safety and sustainability of our freshwater resources (Paerl, 1988; Huisman *et al*., 2005) (Fig. 1). Specifically, CyanoHABs are adept at taking advantage of anthropogenic nutrient over‐enrichment (eutrophication) and hydrologic modifications (e.g. water withdrawal, construction of dams and reservoirs) (Huisman *et al*., 2005; Paerl and Otten, 2013). In addition, climatic changes taking place, including warming, more intense and frequent storms and droughts, favour bloom formation in diverse aquatic environments (Paerl and Huisman, 2009; Paerl *et al*., 2011) (Fig. 2).

There is an urgent need to mitigate global CyanoHAB expansion. As climatic changes are difficult to control on the short term (i.e. years to decades), the ‘knobs we can tweek’ are those modulating nutrient inputs and hydrologic alterations. In addition, algaecides, artificial mixing, flushing and flocculation have been used to temporarily arrest blooms; however, these techniques should be coupled with nutrient input reductions (Paerl *et al*., 2016a,b).

## Nutrients and hydrology: Key controls of CyanoHABs

Among the nutrient elements essential for aquatic plant growth, nitrogen (N) and phosphorus (P) are often most stimulatory, because cellular requirements are high relative to availability. Excessive inputs of both N and P can promote hypereutrophic conditions with chlorophyll *a* (indicator of algal biomass) concentrations exceeding 50 μg l^−1^ and bloom episodes (Huisman *et al*., 2005; Lewis *et al*., 2011; Schindler, 2012). These conditions are especially problematic in shallow lakes and reservoirs in which recent accelerated N and P loading has led to nutrient accumulation in sediments, which represent a ‘bank’ of nutrients that can be regenerated, thus ensuring continued availability. While P‐over‐enrichment is a chronic problem (Schindler, 2012), we are now facing a growing nitrogen ‘glut’, with unprecedented amounts of biologically available forms of N discharged from synthetic N fertilizers, industrial‐scale animal operations, fossil fuel and agricultural atmospheric N emissions, all increasing at alarming rates globally (Galloway *et al*., 2004; Howarth *et al*., 2012). This has led to receiving waters becoming relatively N‐enriched and more eutrophic (Lewis *et al*., 2011; Paerl *et al*., 2016a).

The continuing threat from N‐enhanced eutrophication persists because (i) some N is ‘lost’ by denitrification, creating a continuing demand for N supplies to sustain eutrophication (Paerl *et al*., 2016a), (ii) historically high loads of P are stored in sediments, creating a reservoir for recycled P to sustain eutrophication, and (iii) in nutrient‐impacted eutrophic lakes, N_2_ fixation does not meet ecosystem‐scale N requirements (Paerl and Scott, 2010). Therefore, eutrophication can be further accelerated by increasing external N inputs (Paerl *et al*., 2016a).

A troubling indicator of N‐driven accelerated eutrophication and proliferating CyanoHABs is the global expansion of the non‐N_2_ fixing genus *Microcystis* in our waterways (Paerl *et al*., 2016b). This genus and benthic analogues (*Lyngbya* and *Oscillatoria* spp.) serve as indicators of N‐over‐enrichment (Paerl *et al*., 2016a,b).

Proliferating non‐N_2_ fixing CyanoHABs indicate that N input reductions are needed to stem this troubling trend. However, the potential exists for N_2_ fixing cyanobacterial species to replace non‐N_2_ fixing ones when N inputs are reduced, especially if P remains available to support primary production (Schindler, 2012). Such a scenario is of concern in lakes and reservoirs used for drinking water, fishing and recreational purposes, because members of the toxic non‐N_2_ fixing genus (e.g. *Microcystis*) could be replaced by other noxious N_2_ fixing species (e.g. *Dolichospermum* spp., *Aphanizomenon* spp., *Nodularia* spp.). This possibility should be examined in lakes and reservoirs in which N‐input reductions are being enacted to reduce overall trophic state and CyanoHABs. Recent studies have shown, however, that dual nutrient reduction strategies are needed for many hypereutrophic systems experiencing excessive loading and internal storage of P and N (Lewis *et al*., 2011; Paerl *et al*., 2016b).

### Hydrologic manipulations

In addition to reductions in N and P inputs, hydrologic modifications can modulate CyanoHABs. Artificial mixing of lakes and ponds, by air bubbling or other mixing devices, is used to decrease water column stratification and enhance vertical mixing of the phytoplankton, thereby preventing the formation of surface blooms of buoyant cyanobacteria (Mitrovic *et al*., 2003; Huisman *et al*., 2005). Flushing by increasing water flow through impacted systems reduces water residence time, limiting development of CyanoHABs (Paerl *et al*., 2016b). While these approaches can suppress CyanoHABs, hydrologic changes can be quite expensive and restricted to relatively small water bodies, and freshwater supplies for flushing may be limited. Furthermore, consequences of flushing nutrient‐rich waters to downstream nutrient‐sensitive waterbodies must be assessed.

## Conclusions

I have explored the interactive physical, chemical and biotic factors implicated in the development, proliferation and expansion of CyanoHABs. In addition to their seemingly limitless adaptations to environmental change on both geological and biological time scales, CyanoHABs are impacted by human alterations of aquatic environments. The most notable and controllable alterations include (i) excessive nutrient (especially N and P) inputs and (ii) hydrologic changes, including freshwater diversions, the construction of impoundments such as reservoirs, water use for irrigation, drinking, flood control, all of which affect water residence time or flushing rates.

Effective long‐term management of CyanoHABs must be evaluated on a system‐specific basis, as nutrient‐ and flushing‐bloom thresholds will vary, depending on system geomorphological and hydrologic characteristics. We also must be mindful of the ecological and physiological adaptations that certain taxa possess to circumvent controls derived from our knowledge of these factors. Examples include (i) the ability of N_2_ fixing taxa to exploit N‐limited conditions, (ii) the ability of certain buoyant taxa to counteract mixing aimed at minimizing cyanobacterial dominance, (iii) specific mutualistic and symbiotic associations that cyanobacteria have with other microorganisms, higher plants and animals, which may provide clues as to the roles toxins and other chemical factors play in shaping biotic community structure and function.

## Conflict of Interest

None declared.
